# Propyl 4-amino­benzoate

**DOI:** 10.1107/S2414314626004566

**Published:** 2026-05-07

**Authors:** Alexander J. Gutzwiller, Christine K. F. Hermann, George N. Harakas

**Affiliations:** a PO Box 6949, Radford University, Radford, Virginia 24142, USA

**Keywords:** crystal structure, hydrogen bonding

## Abstract

The structure of the title compound, C_10_H_13_NO_2_, shows the *n*-propyl group to be perpendicular to the remaining part of the mol­ecule; carboxyl­ate-C—O—C—C(*n*-prop­yl) torsion angle = −87.05 (19)°. In the crystal, amine-N—H⋯O(carbon­yl) and weaker amine-N—H⋯N(amine) hydrogen bonding occurs within double layers parallel to the *ab* plane. The hydrogen atoms of the methyl group are disordered over two sets of sites in the ratio 0.89 (3) to 0.11 (3).

## Abstract 2

The title compound C_10_H_13_NO_2_ was synthesized through a Fischer esterification. The crystal structure complements the physical and spectroscopic data collected as part of a guided inquiry undergraduate Organic Chemistry laboratory experiment.

## Graphical abstract

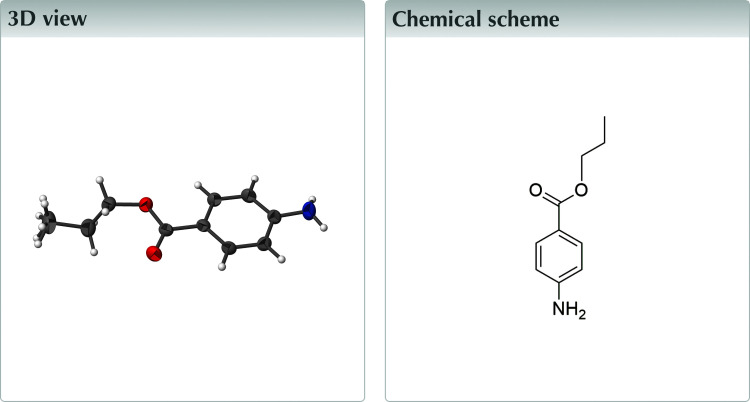

## Structure description

The reaction of 4-amino­benzoic acid with 1-propanol yielded the title compound *n*-propyl 4-amino­benzoate (risocaine). Risocaine, its isomer, isopropyl 4-amino­benzoate, and amines with similar structures have medical applications such as pain relievers (Priyanka *et al.*, 2022 ▸).

The crystal structure we report complements the physical and spectroscopic data collected as part of a guided inquiry undergraduate Organic Chemistry laboratory experiment (Hermann *et al.*, 2026 ▸). In this guided inquiry experiment, students synthesized and characterized solid esters, selected for ease of purification and handling. Students were provided with a list of possible carb­oxy­lic acids and alcohols but not the specific reactants assigned to each group. After synthesis, they compared the observed melting point range of their product to a reference table. Furthermore, students recorded the IR,^1^H NMR, and ^13^C NMR spectra of their product to confirm their conclusions from the melting point range.

**Figure 1:**
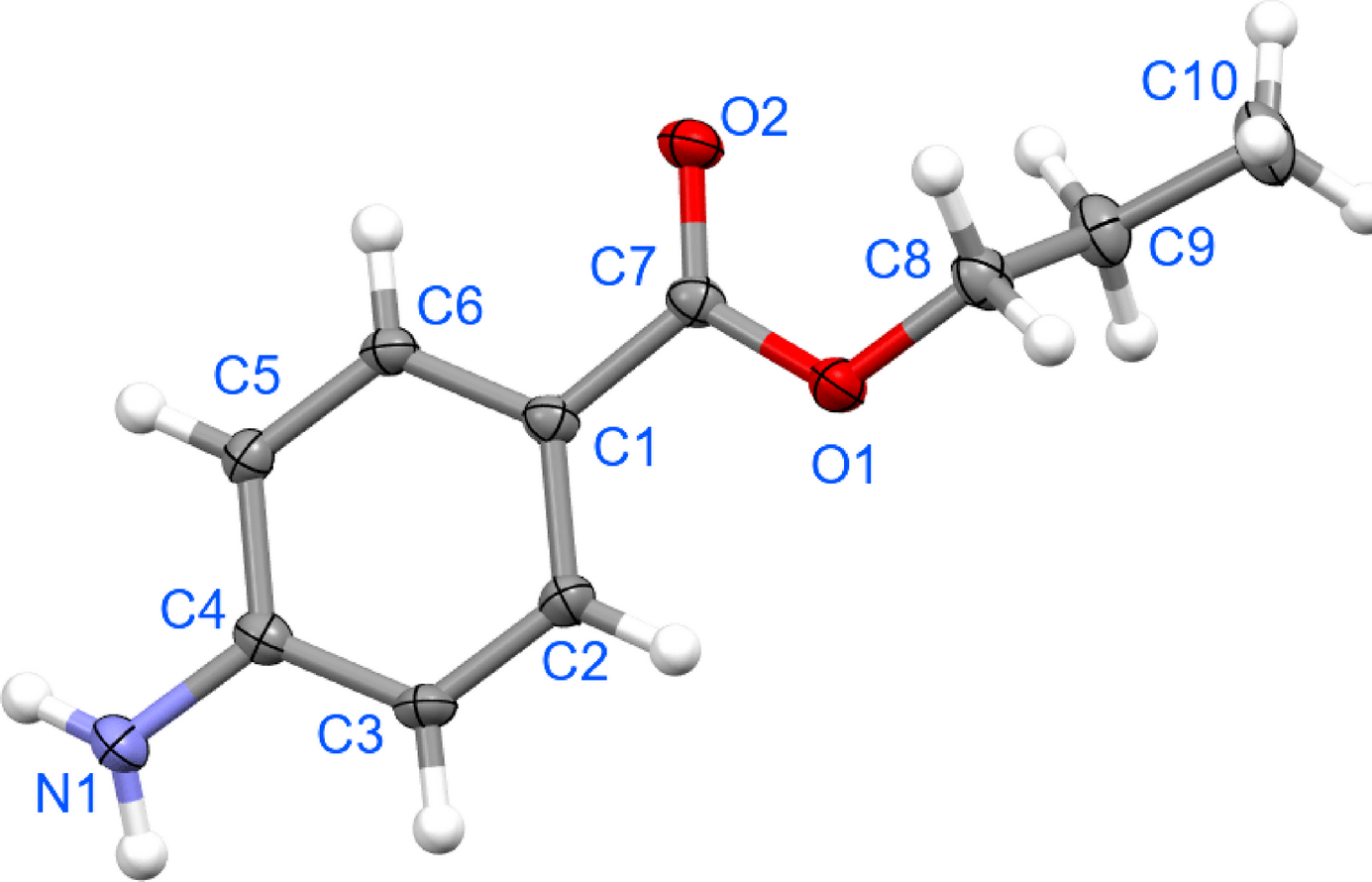
The mol­ecular structure of the title compound showing displacement ellipsoids at the 30% probability level. Only the major component of the disordered hydrogen atoms on C10 are shown.

Referring to Fig. 1 ▸, the C9 atom of the *n*-propyl group is close to perpendicular to the carboxyl­ate residue as seen in the C7—O1—C8—C9 torsion angle of −87.05 (19)°. The equivalent torsion angles for one of the terminal –CH_3_ groups in isopropyl 4-amino­benzoate (two independent mol­ecules) are −77.8 (4) and −86.5 (4)°, while the other –CH_3_ groups approach coplanarity, *i.e*. 159.5 (3) and 152.5 (4)° (Priyanka *et al.*, 2022 ▸). The carboxyl­ate [the C2—C1—C7—O2 torsion angle = 176.86 (17)°] and amine [C2—C3—C4—N1 = 174.94 (17)°] groups are close to coplanar to the benzene ring to which they are connected.

**Figure 2:**
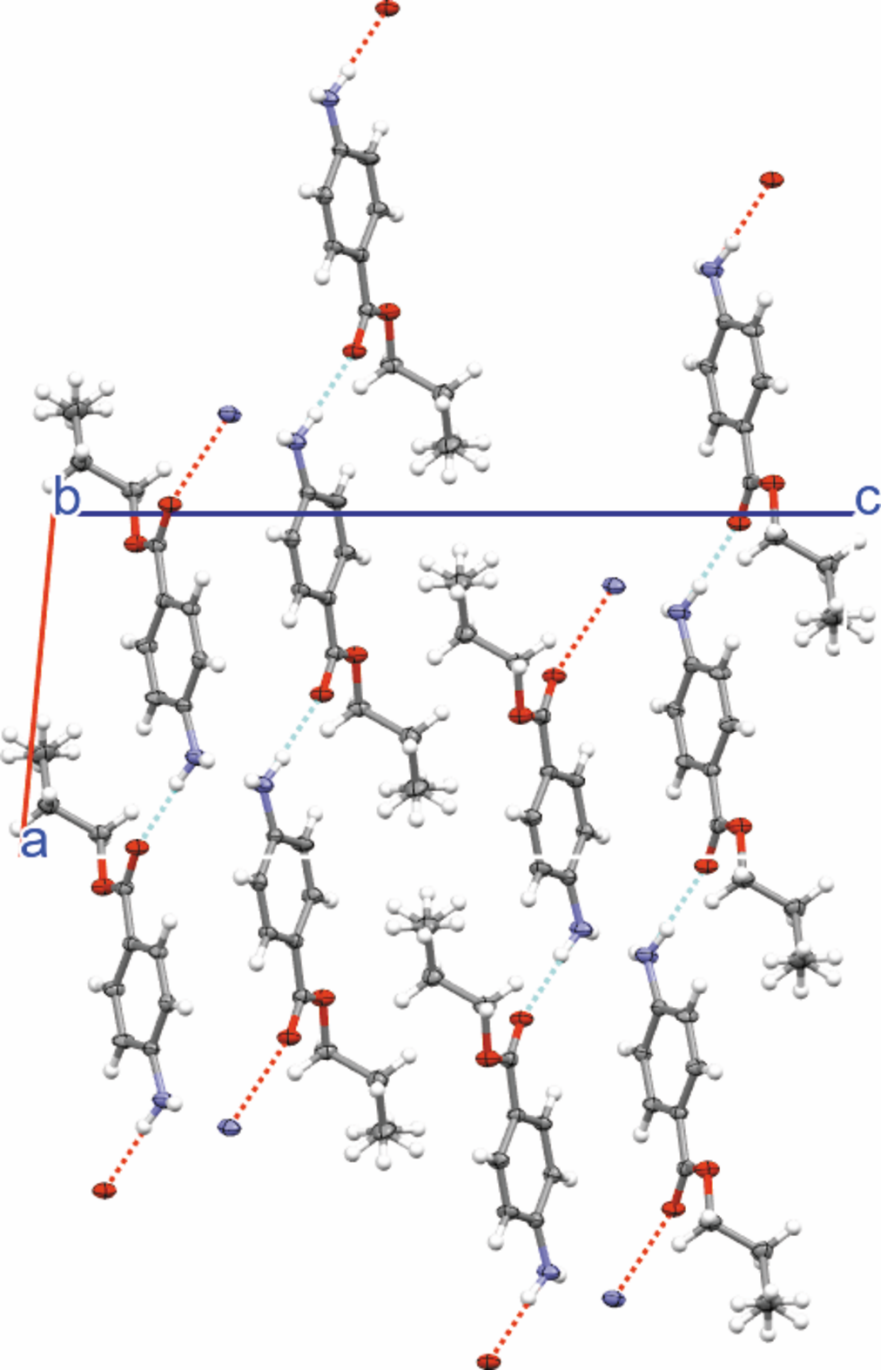
Crystal packing viewed along the *b* axis. Hydrogen-bonding interactions are shown as dashed lines.

The mol­ecular packing (Fig. 2 ▸) shows hydrogen-bonding inter­actions between the amine functional group and the carbonyl group of the ester and weaker amine-N—H⋯N(amine) hydrogen bonding; distances and angle are listed in Table 1 ▸. The hydrogen bonding occurs within double layers that stack along the *c* axis.

## Synthesis and crystallization

**Figure 3:**
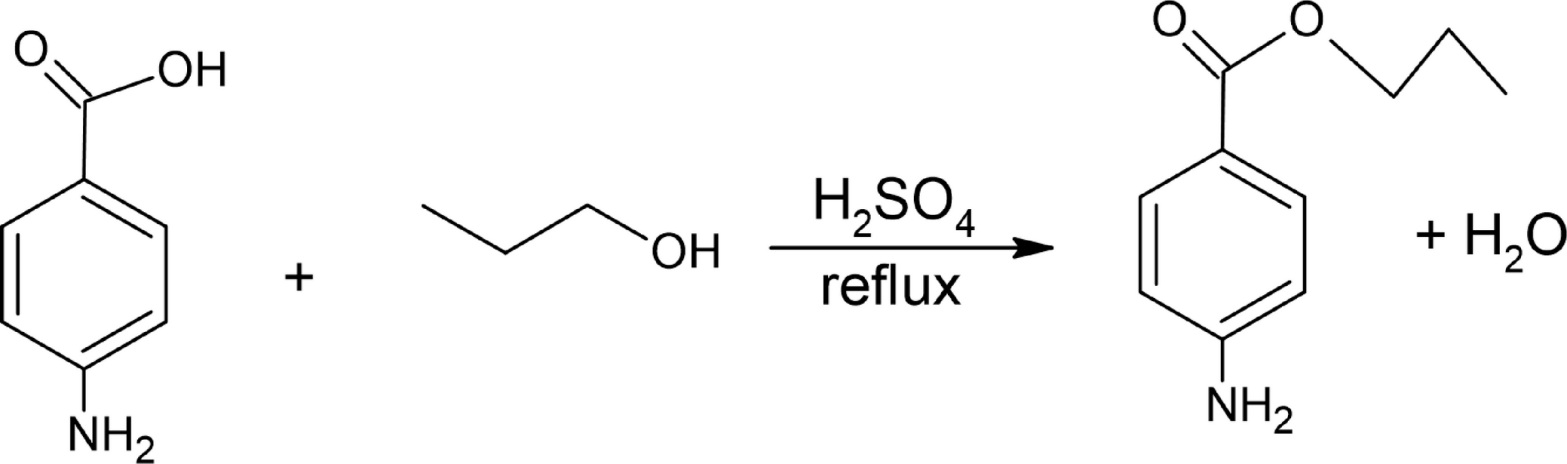
Reaction scheme for the title compound.

Referring to Fig. 3 ▸, the title compound was synthesized through a Fischer esterification. A mixture of 4-amino­benzoic acid (1.5 g), 1-propanol (10 ml), and concentrated sulfuric acid (1 ml) was refluxed in a 50 ml boiling flask for 1 h. The reaction mixture was allowed to cool; a solution of 10% sodium carbonate was added until a pH of 8 was obtained. The solution was chilled in an ice bath until a solid product was formed. The solid was isolated by vacuum filtration.

The yield was 0.864 grams (57.0%) with a melting point of 72.7°C, which compared to a literature value of 73–75°C. The IR and NMR spectra confirmed the structure (Hermann *et al.*, 2026 ▸).

X-ray quality crystals were produced by dissolving the product into methanol, followed by adding an equal volume of hexa­nes. The solvent was allowed to evaporate over several days. A single-crystal was coated with NVH oil and mounted on a MiTeGen loop then cooled to −40 °C for data collection.

## Refinement

Crystal data, data collection, and structure refinement details are summarized in Table 2 ▸. The hydrogen atoms on the methyl-C10 atom are disordered over two positions and refined to a 0.89 (3) to 0.11 (3) occupancy ratio.

## Supplementary Material

Crystal structure: contains datablock(s) I. DOI: 10.1107/S2414314626004566/tk4124sup1.cif

Structure factors: contains datablock(s) I. DOI: 10.1107/S2414314626004566/tk4124Isup2.hkl

Supporting information file. DOI: 10.1107/S2414314626004566/tk4124Isup3.cml

CCDC reference: 2550860

Additional supporting information:  crystallographic information; 3D view; checkCIF report

## Figures and Tables

**Table 1:** Hydrogen-bond geometry (Å, °)

*D*—H⋯*A*	*D*—H	H⋯*A*	*D*⋯*A*	*D*—H⋯*A*
N1—H1*A*⋯N1^i^	0.88 (2)	2.55 (2)	3.397 (3)	162 (2)
N1—H1*B*⋯O2^ii^	0.88 (2)	2.09 (2)	2.964 (2)	173 (2)

Symmetry codes: (i) 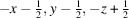; (ii) 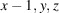.

**Table 2:** Experimental details

Crystal data	
Chemical formula	C_10_H_13_NO_2_
*M*_r_	179.21
Crystal system, space group	Monoclinic, *P*2_1_/*n*
Temperature (K)	233
*a*, *b*, *c* (Å)	8.5011 (3), 5.8300 (2), 19.7631 (8)
β (°)	95.453 (2)
*V* (Å^3^)	975.05 (6)
*Z*	4
Radiation type	Mo *K*α
μ (mm^−1^)	0.09
Crystal size (mm)	0.41 × 0.21 × 0.21

Data collection	
Diffractometer	Bruker D8
Absorption correction	Multi-scan (*SADABS*; Krause *et al.*, 2015 ▸)
*T*_min_, *T*_max_	0.966, 0.982
No. of measured, independent and observed [*I* > 2σ(*I*)] reflections	37794, 2427, 2283
*R*_int_	0.039
(sin θ/λ)_max_ (Å^−1^)	0.668

Refinement	
*R*[*F*^2^ > 2σ(*F*^2^)], *wR*(*F*^2^), *S*	0.069, 0.159, 1.17
No. of reflections	2427
No. of parameters	126
No. of restraints	5
H-atom treatment	H atoms treated by a mixture of independent and constrained refinement
Δρ_max_, Δρ_min_ (e Å^−3^)	0.39, −0.27

Computer programs: *APEX3* and *SAINT* (Bruker, 2019 ▸), *SHELXT2018/2* (Sheldrick, 2015*a* ▸), *SHELXL2019/2* (Sheldrick, 2015*b* ▸) and *ShelXle* (Hübschle *et al.*, 2011 ▸).

## full crystallographic data

### Propyl 4-aminobenzoate Crystal data

**Table:**

C_10_H_13_NO_2_	*F*(000) = 384
*M**_r_* = 179.21	*D*_x_ = 1.221 Mg m^−^^3^
Monoclinic, *P*2_1_/*n*	Mo *K*α radiation, λ = 0.71073 Å
*a* = 8.5011 (3) Å	Cell parameters from 9986 reflections
*b* = 5.8300 (2) Å	θ = 3.7–28.3°
*c* = 19.7631 (8) Å	µ = 0.09 mm^−^^1^
β = 95.453 (2)°	*T* = 233 K
*V* = 975.05 (6) Å^3^	Block, colourless
*Z* = 4	0.41 × 0.21 × 0.21 mm

### Propyl 4-aminobenzoate Data collection

**Table:**

Bruker D8 diffractometer	2427 independent reflections
Radiation source: sealed tube	2283 reflections with *I* > 2σ(*I*)
Flat graphite monochromator	*R*_int_ = 0.039
Detector resolution: 7.391 pixels mm^-1^	θ_max_ = 28.3°, θ_min_ = 3.7°
ω and φ scans	*h* = −11→11
Absorption correction: multi-scan (SADABS; Krause *et al.*, 2015)	*k* = −7→7
*T*_min_ = 0.966, *T*_max_ = 0.982	*l* = −26→26
37794 measured reflections	

### Propyl 4-aminobenzoate Refinement

**Table:**

Refinement on *F*^2^	Primary atom site location: Intrinsic Phasing
Least-squares matrix: full	Hydrogen site location: mixed
*R*[*F*^2^ > 2σ(*F*^2^)] = 0.069	H atoms treated by a mixture of independent and constrained refinement
*wR*(*F*^2^) = 0.159	*w* = 1/[σ^2^(*F*_o_^2^) + (0.0557*P*)^2^ + 0.6285*P*] where *P* = (*F*_o_^2^ + 2*F*_c_^2^)/3
*S* = 1.17	(Δ/σ)_max_ < 0.001
2427 reflections	Δρ_max_ = 0.39 e Å^−^^3^
126 parameters	Δρ_min_ = −0.27 e Å^−^^3^
5 restraints	

### Propyl 4-aminobenzoate Special details

**Table:**

Geometry. All esds (except the esd in the dihedral angle between two l.s. planes) are estimated using the full covariance matrix. The cell esds are taken into account individually in the estimation of esds in distances, angles and torsion angles; correlations between esds in cell parameters are only used when they are defined by crystal symmetry. An approximate (isotropic) treatment of cell esds is used for estimating esds involving l.s. planes.

### Propyl 4-aminobenzoate Fractional atomic coordinates and isotropic or equivalent isotropic displacement parameters (Å^2^)

**Table:**

	*x*	*y*	*z*	*U*_iso_*/*U*_eq_	Occ. (<1)
O1	0.41184 (15)	0.7084 (2)	0.39339 (8)	0.0407 (4)	
O2	0.52677 (15)	0.3933 (2)	0.35610 (7)	0.0420 (4)	
N1	−0.21045 (19)	0.1716 (3)	0.29235 (10)	0.0450 (4)	
H1A	−0.220 (3)	0.028 (3)	0.2790 (13)	0.068*	
H1B	−0.287 (2)	0.229 (4)	0.3145 (13)	0.068*	
C1	0.24626 (19)	0.4106 (3)	0.35136 (8)	0.0300 (4)	
C2	0.1139 (2)	0.5332 (3)	0.36787 (10)	0.0362 (4)	
H2	0.127429	0.673133	0.391334	0.043*	
C3	−0.0363 (2)	0.4507 (3)	0.35001 (10)	0.0389 (4)	
H3	−0.124141	0.534562	0.361780	0.047*	
C4	−0.0597 (2)	0.2444 (3)	0.31471 (9)	0.0324 (4)	
C5	0.0731 (2)	0.1194 (3)	0.29957 (9)	0.0327 (4)	
H5	0.059756	−0.022164	0.276956	0.039*	
C6	0.2229 (2)	0.2018 (3)	0.31749 (9)	0.0320 (4)	
H6	0.310763	0.116125	0.306731	0.038*	
C7	0.4082 (2)	0.4970 (3)	0.36686 (9)	0.0313 (4)	
C8	0.5655 (2)	0.8188 (3)	0.40383 (11)	0.0403 (4)	
H8A	0.551542	0.985495	0.400907	0.048*	
H8B	0.629878	0.771708	0.367673	0.048*	
C9	0.6508 (2)	0.7587 (4)	0.47167 (11)	0.0471 (5)	
H9A	0.680761	0.596304	0.472127	0.057*	
H9B	0.580694	0.783509	0.507594	0.057*	
C10	0.7985 (3)	0.9069 (5)	0.48517 (14)	0.0621 (7)	
H10A	0.866055	0.885591	0.448792	0.093*	0.89 (3)
H10B	0.855180	0.862316	0.528067	0.093*	0.89 (3)
H10C	0.768012	1.066875	0.487229	0.093*	0.89 (3)
H10D	0.793444	0.990931	0.527267	0.093*	0.11 (3)
H10E	0.804318	1.014205	0.447992	0.093*	0.11 (3)
H10F	0.891486	0.809646	0.488829	0.093*	0.11 (3)

### Propyl 4-aminobenzoate Atomic displacement parameters (Å^2^)

**Table:**

	*U*^11^	*U*^22^	*U*^33^	*U*^12^	*U*^13^	*U*^23^
O1	0.0293 (6)	0.0326 (7)	0.0598 (9)	0.0002 (5)	0.0014 (6)	−0.0065 (6)
O2	0.0275 (6)	0.0401 (8)	0.0582 (9)	0.0056 (5)	0.0037 (6)	−0.0025 (6)
N1	0.0287 (8)	0.0482 (10)	0.0585 (11)	−0.0060 (7)	0.0061 (7)	−0.0119 (9)
C1	0.0271 (8)	0.0291 (8)	0.0336 (8)	0.0033 (6)	0.0022 (6)	0.0025 (7)
C2	0.0315 (8)	0.0311 (9)	0.0457 (10)	0.0040 (7)	0.0017 (7)	−0.0074 (8)
C3	0.0270 (8)	0.0363 (10)	0.0538 (11)	0.0090 (7)	0.0054 (7)	−0.0077 (8)
C4	0.0273 (8)	0.0354 (9)	0.0344 (8)	0.0002 (7)	0.0035 (6)	0.0025 (7)
C5	0.0347 (9)	0.0290 (8)	0.0345 (8)	0.0001 (7)	0.0040 (7)	−0.0042 (7)
C6	0.0278 (8)	0.0311 (9)	0.0375 (9)	0.0057 (7)	0.0051 (6)	−0.0016 (7)
C7	0.0295 (8)	0.0299 (8)	0.0344 (8)	0.0032 (7)	0.0021 (6)	0.0042 (7)
C8	0.0355 (9)	0.0317 (9)	0.0534 (11)	−0.0063 (8)	0.0025 (8)	0.0034 (8)
C9	0.0427 (11)	0.0492 (12)	0.0489 (11)	−0.0090 (9)	0.0010 (9)	0.0010 (9)
C10	0.0493 (13)	0.0730 (17)	0.0619 (14)	−0.0224 (12)	−0.0059 (10)	−0.0058 (13)

### Propyl 4-aminobenzoate Geometric parameters (Å, º)

**Table:**

O1—C7	1.338 (2)	C2—C3	1.379 (3)
O1—C8	1.453 (2)	C3—C4	1.395 (3)
O2—C7	1.212 (2)	C4—C5	1.400 (2)
N1—C4	1.382 (2)	C5—C6	1.376 (2)
C1—C6	1.394 (2)	C8—C9	1.504 (3)
C1—C2	1.398 (2)	C9—C10	1.527 (3)
C1—C7	1.470 (2)		
			
C7—O1—C8	116.80 (14)	C3—C4—C5	118.45 (16)
C6—C1—C2	118.51 (16)	C6—C5—C4	120.56 (16)
C6—C1—C7	119.17 (15)	C5—C6—C1	121.01 (16)
C2—C1—C7	122.30 (16)	O2—C7—O1	122.63 (16)
C3—C2—C1	120.55 (17)	O2—C7—C1	124.79 (17)
C2—C3—C4	120.88 (16)	O1—C7—C1	112.57 (14)
N1—C4—C3	120.56 (16)	O1—C8—C9	111.97 (16)
N1—C4—C5	120.92 (17)	C8—C9—C10	110.04 (19)

### Propyl 4-aminobenzoate Hydrogen-bond geometry (Å, º)

**Table:**

*D*—H···*A*	*D*—H	H···*A*	*D*···*A*	*D*—H···*A*
N1—H1*A*···N1^i^	0.88 (2)	2.55 (2)	3.397 (3)	162 (2)
N1—H1*B*···O2^ii^	0.88 (2)	2.09 (2)	2.964 (2)	173 (2)

Symmetry codes: (i) −*x*−1/2, *y*−1/2, −*z*+1/2; (ii) *x*−1, *y*, *z*.
